# Point-of-Care Ultrasound in the Diagnosis of Hematoma Mimicking Deep Vein Thrombosis: A Case Report

**DOI:** 10.7759/cureus.93401

**Published:** 2025-09-28

**Authors:** Sunil Sharan, Latif Rahman, Md Ariful Islam, Vickie Wong, Md Samiul Islam, Asif Swapnil, Adarsh Bhowon

**Affiliations:** 1 Acute Medicine, Leicester Royal Infirmary Hospital, Leicester, GBR

**Keywords:** anticoagulation, bedside ultrasound, deep vein thrombosis, lower limb swelling, muscle hematoma, point-of-care ultrasound

## Abstract

Deep vein thrombosis (DVT) is a common yet potentially serious condition encountered by acute care physicians. However, it can present with symptoms that mimic other conditions, such as spontaneous muscle hematoma, making accurate differentiation essential. We report the case of a 38-year-old man who developed right leg swelling and tenderness seven days after an uncomplicated femoral access coronary angiogram. Clinical suspicion for DVT was supported by elevated D-dimer levels, and enoxaparin therapy was initiated. However, point-of-care ultrasound (POCUS) identified a large muscle hematoma. Orthopedic assessment ruled out compartment syndrome, and a formal Doppler ultrasound of the lower limb confirmed the diagnosis. Anticoagulation was discontinued, and a follow-up scan was scheduled to monitor hematoma resolution. This case underscores the critical role of bedside imaging in acute presentations where clinical findings may be misleading. POCUS bridged the gap between clinical suspicion and definitive diagnosis, playing a pivotal role in guiding patient management.

## Introduction

Lower limb swelling and pain are common presentations in acute medicine and ambulatory care units, with deep vein thrombosis (DVT) being a primary concern due to its potentially life-threatening complications, such as pulmonary embolism [1,2]. Anticoagulation is often initiated promptly when clinical suspicion is high and D-dimer levels are elevated [3]. However, muscle hematoma can mimic DVT on clinical assessment, potentially leading to inappropriate management and patient harm [4].

Point-of-care ultrasound (POCUS) has emerged as a transformative diagnostic tool in modern medical care, providing immediate bedside imaging that enhances clinical decision-making [5]. In situations where conventional radiology imaging is delayed or unavailable, POCUS allows rapid assessment of complex and potentially serious conditions, aiding patient management and improving outcomes [6,7].

In this report, we describe a case of a young patient who presented to the emergency department with DVT-like clinical features and was started on empirical anticoagulation while awaiting formal Doppler imaging. Bedside POCUS revealed a deep muscle hematoma, allowing safe cessation of empirical anticoagulation and avoidance of complications, highlighting POCUS’s pivotal role in acute care.

Although POCUS is increasingly recognized as a valuable modality for differentiating DVT from alternative pathologies, this case illustrates a unique diagnostic challenge. The patient presented with a clinical picture highly suggestive of venous thrombosis; however, bedside ultrasound identified an intramuscular hematoma located anatomically distant from the recent angiogram puncture site. This discordance between the anticipated complication and the actual pathology increased the risk of misdiagnosis. Immediate POCUS not only excluded DVT but also provided a definitive alternative diagnosis, thereby preventing inappropriate anticoagulation. This case underscores the importance of structured ultrasound protocols in acute medicine and demonstrates how timely bedside imaging can enhance diagnostic accuracy, safeguard patient outcomes, and optimize healthcare resource use.

## Case presentation

A 38-year-old gentleman re-presented to the emergency department with new-onset swelling and tenderness in his right leg. He had been discharged a week earlier following a coronary angiogram. He denied any recent trauma, prolonged immobility, or long-haul travel. There was no personal or family history of thromboembolic disease or bleeding disorders.

On physical examination, there was localized mild tenderness along the deep venous system of the right leg, accompanied by mild visible ecchymosis. There were no signs of erythema, warmth, or increased compartmental pressure. Peripheral pulses were palpable bilaterally. Vital signs were within normal limits, and the patient was hemodynamically stable.

Given the recent vascular procedure and unilateral leg swelling, DVT was suspected. A D-dimer test was performed and found to be elevated, supporting clinical concern. Therapeutic low molecular weight heparin (enoxaparin) was initiated empirically in accordance with local protocols. However, the presence of visible bruising prompted further evaluation with bedside POCUS at the time of presentation.

**Figure 1:**
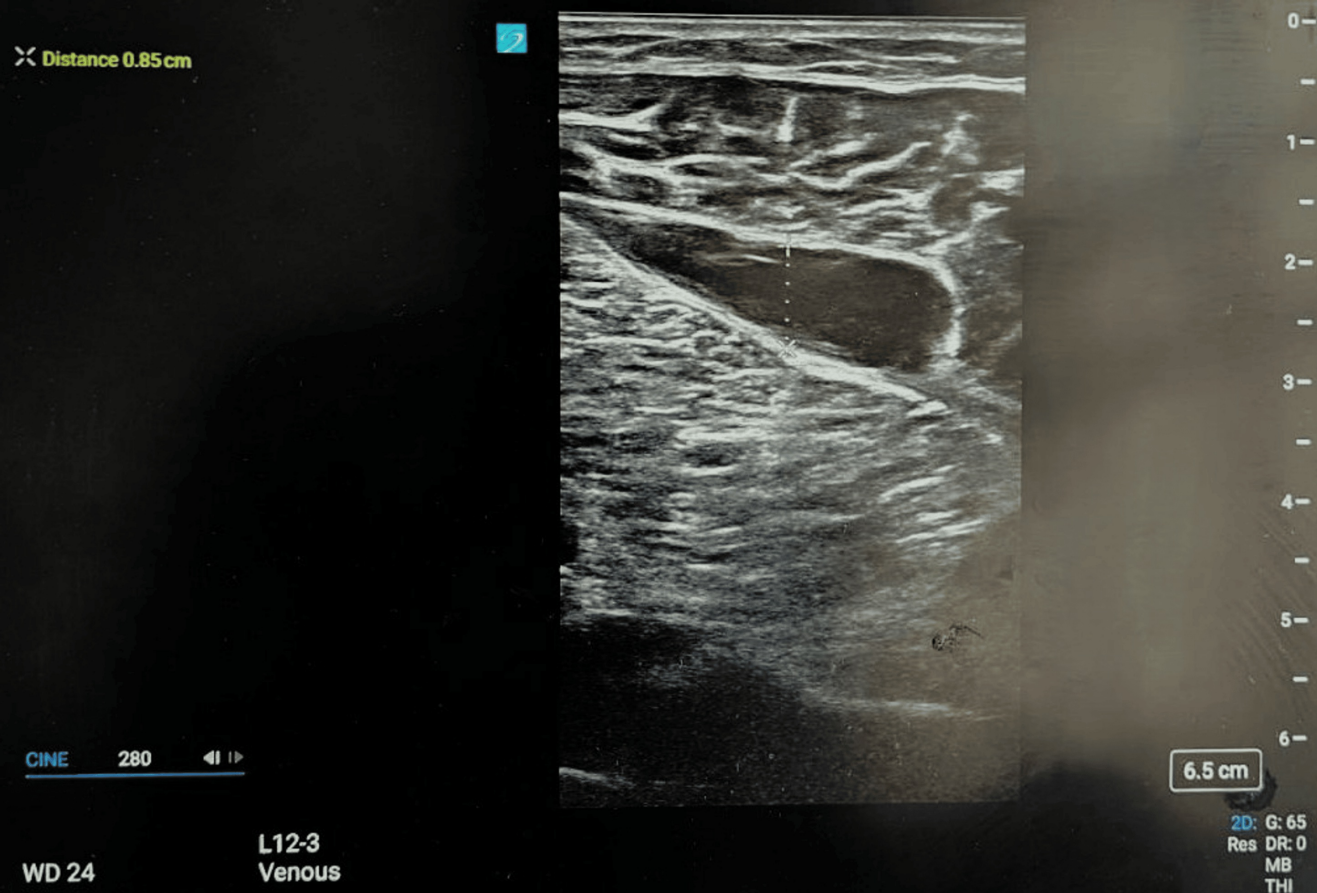
POCUS image showing a well-defined intramuscular hematoma in the right calf POCUS, point-of-care ultrasound

A high-frequency linear transducer was used, with the patient positioned supine and the leg externally rotated. A structured compression ultrasound protocol was applied at the common femoral, superficial femoral, and popliteal veins, supplemented by color Doppler when compressibility was uncertain. Bedside POCUS revealed no evidence of thrombus in the femoral or popliteal veins but identified a well-defined hematoma measuring approximately 6 cm × 5 cm (Figure 1).

**Figure 2:**
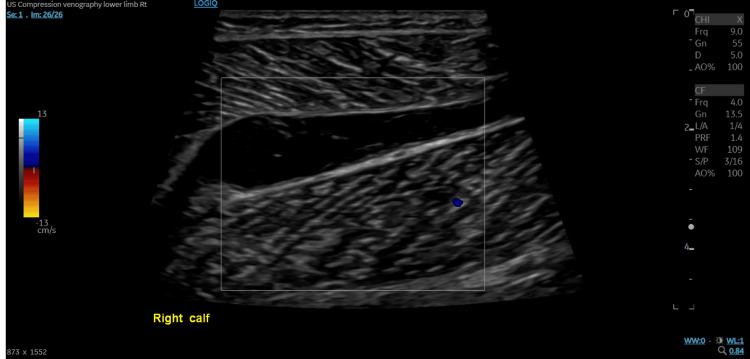
Ultrasound compression venography of the right calf demonstrating an avascular, cystic area with features suggestive of hemorrhage

The patient was urgently reviewed by the orthopedic team, who clinically ruled out compartment syndrome. A formal ultrasound compression venography of the right lower limb was performed, showing that the right common femoral, femoral, and popliteal veins were patent and compressible. The visualized deep calf veins were also patent and compressible. A 67 mm × 14 mm × 30 mm avascular, cystic area was identified within the right calf, containing low-level echoes suggestive of hemorrhage (Figure 2). The differential diagnosis was a hematoma, and DVT was definitively excluded.

**Figure 3:**
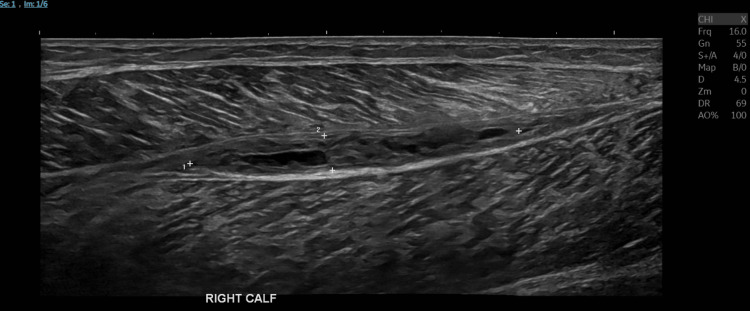
Repeat ultrasound compression venography of the right calf showing a reduction in the size of the hematoma

In view of the hematoma, enoxaparin was discontinued. The patient had received only a single therapeutic dose of enoxaparin. A repeat ultrasound performed two weeks later demonstrated a significant reduction in the size of the hematoma, measuring 57 mm × 6 mm × 14 mm (Figure 3). 

## Discussion

This case illustrates the diagnostic value of POCUS in differentiating DVT from other causes of unilateral leg swelling, such as a hematoma. In post-procedural patients, particularly those who have undergone recent vascular interventions, hematomas can closely mimic DVT both clinically and biochemically [8,9]. Reliance on elevated D-dimer levels alone may be misleading, as they are nonspecific and frequently elevated in the post-intervention setting [10,11].

The timely use of bedside POCUS in this case prevented inappropriate anticoagulation, which could have exacerbated the hematoma and increased the risk of complications, including bleeding and compartment syndrome [12,13]. POCUS has demonstrated high sensitivity and specificity for detecting proximal DVT and is now widely adopted as a frontline diagnostic tool in emergency and acute care settings [14,15]. Clinicians trained in limited compression ultrasonography can reliably evaluate for thrombus at the femoral and popliteal levels, with diagnostic performance comparable to formal Doppler studies [14,16].

Furthermore, POCUS offers the additional advantage of detecting alternative diagnoses. In this case, it revealed a large, well-defined intramuscular hematoma, prompting a change in clinical management and the cessation of anticoagulation. Similar reports have highlighted the utility of POCUS in identifying soft tissue abnormalities, including muscle hematomas and cysts, that can closely mimic DVT [17,18]. Localized swelling without significant risk factors for venous thromboembolism or pain disproportionate to physical findings should raise suspicion for alternative diagnoses such as hematoma or abscess. POCUS is particularly valuable in these scenarios, as it allows simultaneous exclusion of DVT and early identification of mimics, especially when combined with clinical risk stratification tools such as the Wells score and D-dimer testing.

In addition to preventing unnecessary anticoagulation and prolonged admission, early use of POCUS in this case had broader economic implications. By avoiding inappropriate anticoagulation, it mitigated the risk of bleeding complications, which are associated with significant morbidity and high treatment costs. Bedside ultrasound also reduced dependence on radiology services, preserving imaging capacity for patients requiring urgent investigations. Furthermore, by facilitating earlier discharge, POCUS contributed to improved patient flow and bed availability in an already pressured acute care environment.

Integrating POCUS into standard diagnostic protocols for lower limb swelling may enhance diagnostic accuracy, reduce unnecessary treatment, and improve patient safety, particularly in ambulatory or resource-limited settings. In this instance, its use enabled early recognition of a non-thrombotic pathology, guiding safer and more effective management.

## Conclusions

This case demonstrates the pivotal role of POCUS in the rapid evaluation of post-procedural leg swelling. Beyond aiding accurate diagnosis, POCUS prevented the continuation of unnecessary and potentially harmful anticoagulation therapy. It underscores the importance of considering alternative causes of leg swelling following vascular procedures and highlights the value of bedside imaging in guiding timely and appropriate clinical decision-making.
